# The Effect of Body Adiposity and Alcohol Consumption on Serum Uric Acid: A Quantile Regression Analysis Based on the China National Health Survey

**DOI:** 10.3389/fnut.2021.724497

**Published:** 2022-01-17

**Authors:** Huijing He, Li Pan, Xiaolan Ren, Dingming Wang, Jianwei Du, Ze Cui, Jingbo Zhao, Hailing Wang, Xianghua Wang, Feng Liu, Lize Pa, Xia Peng, Ye Wang, Chengdong Yu, Guangliang Shan

**Affiliations:** ^1^Department of Epidemiology and Statistics, Institute of Basic Medical Sciences, Chinese Academy of Medical Sciences & School of Basic Medicine, Peking Union Medical College, Beijing, China; ^2^Department of Chronic and Noncommunicable Disease Prevention and Control, Gansu Provincial Center for Disease Control and Prevention, Lanzhou, China; ^3^Department of Chronic and Noncommunicable Disease Prevention and Control, Guizhou Provincial Center for Disease Control and Prevention, Guiyang, China; ^4^Department of Chronic and Noncommunicable Disease Prevention and Control, Hainan Provincial Center for Disease Control and Prevention, Haikou, China; ^5^Department of Chronic and Noncommunicable Disease Prevention and Control, Hebei Provincial Center for Disease Control and Prevention, Shijiazhuang, China; ^6^Department of Epidemiology and Statistics, School of Public Health, Harbin Medical University, Harbin, China; ^7^Department of Chronic and Noncommunicable Disease Prevention and Control, Inner Mongolia Autonomous Region Center for Disease Control and Prevention, Hohhot, China; ^8^Integrated Office, Institute of Biomedical Engineering, Chinese Academy of Medical Sciences, Peking Union Medical College, Tianjin, China; ^9^Department of Chronic and Noncommunicable Disease Prevention and Control, Shaanxi Provincial Center for Disease Control and Prevention, Xi'an, China; ^10^Department of Chronic and Noncommunicable Disease Prevention and Control, Xinjiang Uyghur Autonomous Region Center for Disease Control and Prevention, Urumqi, China; ^11^Department of Chronic and Noncommunicable Disease Prevention and Control, Yunnan Provincial Center for Disease Control and Prevention, Kunming, China

**Keywords:** uric acid, quantile regression, body mass index, body fat percentage, alcohol consumption

## Abstract

Adiposity and alcohol consumption are reported to be associated with a higher level of serum uric acid (SUA), but whether their effect differs on SUA percentile distribution is still unclear. In this study, we aimed to investigate how alcohol intake and body fat percentage (%BF) integrated with body mass index (BMI) influence the distribution of SUA in Chinese adults. Data from the China National Health Survey (CNHS) which included adults from 10 provinces of China were used (*n* = 31,746, aged 20–80 years, 40% male). %BF and BMI were integrated into eight expanded body composition groups to understand how excess body adiposity affects the distribution of SUA in the populational level. Self-report alcohol intake information was collected by face-to-face questionnaire interview. Quantile regression (QR) was used to analyze the data. We found that adiposity and alcohol consumption were associated with SUA, especially at the upper percentile in both sexes. In obese men, the QR coefficients at the 75th and 95th percentiles were 74.0 (63.1–84.9) and 80.9 (52.5–109.3) μmol/L, respectively. The highest quartile of %BF in men had a 92.6 (79.3–105.9) μmol/L higher SUA levels at its 95th percentile than the 5th quartile (*p* < 0.001). Compared with normal or underweight with the lowest %BF group (NWBF1), the obesity-highest %BF group (OBBF4) had the strongest positive effect on SUA, especially at the higher percentile of SUA. In BMI-defined normal or underweight participants, a higher quartile of %BF had greater effect size in all SUA percentiles. In men, current alcohol drinking had the strongest effect at the 95th percentile of SUA (QR coefficient: 31.8, with 95% CI: 22.6–41.0) comparing with 14.5, 95% CI of 8.4 to 20.6 in the 5th SUA percentile. High risk of alcohol consumption had a greater effect on SUA, especially in the higher SUA percentile. The observation of stronger association at the higher percentile of SUA suggests that decreasing body adiposity and alcohol intake at the populational level may shift the upper tails of the SUA distributions to lower values, thereby reducing the incidence of hyperuricemia.

## Introduction

The serum uric acid (SUA), the end product of purine metabolism, has been found to be associated with cardiovascular, metabolism diseases, and mortality (1–3). The prevalence of hyperuricemia has been increasing in recent decades, of which the upper percentiles of SUA are of interest in the context of hyperuricemia and other risk diseases (4–7). Adiposity and alcohol consumption are reported to be associated with a higher level of SUA (8, 9), but whether their effect differs on SUA percentile distribution is still unclear.

Statistical methods that extend beyond the analysis of the mean of SUA are needed to build upon previous studies (10). Quantile regression (QR) is a statistical method that can model any percentile of a continuous variable, which facilitates investigators to explore how risk factors affect the shape of SUA. Specifically, this method does not classify subjects into hyperuricemia or non-hyperuricemia groups, which has two drawbacks (11): all participants within a category may be homogeneous, and (2) participants at category boundaries may be very different rather than very similar.

Traditional methods for modeling SUA are based on the conditional mean. However, the upper conditional quantiles are more critical from a public health perspective. Previous studies using traditional statistical analysis methods did not manage to explore how the association of body adiposity and alcohol consumption differs through percentile in the distribution of SUA level. Moreover, the traditional conditional-mean modeling is more sensitive to extreme outliers. Therefore, to address the gap related to adiposity, alcohol consumption, SUA, and the focus on modeling just the mean of SUA, based on a representative national health survey data in China [the China National Health Survey (CNHS)], we analyzed the data using QR method. We aim to understand the effect of body composition and alcohol consumption on the SUA frequency distributions in Chinese adults in mainland China.

## Methods

### Study Design and Participants

As previously described (12), the CNHS data involved adults, and used standardized methods to perform questionnaire interviews and physical examinations from 2012 to 2017. Briefly, using a stratified, multistage cluster sampling method, individuals aged 20–80 years, who had lived in their current residence for at least 1 year, were enrolled from ten provinces of China. Individuals with severe mental or physical illness, pregnant women, or military personnel with active service were excluded. The final sample was restricted to Han ethnic participants who underwent SUA tests.

The study has been carried out in accordance with the Declaration of Helsinki. Ethical approval was obtained from the Bioethical Committee of Institute of Basic Medical Sciences, Chinese Academy of Medical Sciences (No. 029-2013). All participants provided written informed consent before the survey.

### Measurement of SUA and Risk Factors of Interest

Venous blood samples were drawn after an overnight fast. Separated plasma or serum was frozen in aliquots and stored at −80°C until thawed for the first time for the analyses. SUA was measured by oxidization with the specific enzyme uricase on a chemistry analyzer (ROCHE Cobas8000C701, USA).

Adiposity was assessed by body mass index (BMI) and body fat percentage (%BF). Height was measured to the nearest 0.1 cm using a fixed stadiometer. Weight and %BF were measured by a body composition analyzer (TANITA BC-420, Japan), with the accuracy on a decimal level. BMI was calculated as kg/m^2^. Based on the World Health Organization (WHO)'s definition of overweight and obesity, underweight was defined as BMI < 18.5 kg/m^2^, normal weight was BMI between 18.5 and < 25 kg/m^2^, overweight was BMI ≥ 25 kg/m^2^ but < 30 kg/m^2^, and obesity was defined as BMI ≥ 30 kg/m^2^ (13). %BF level was divided into four groups: based on the quartile (< *P*_25_, *P*_25−49_, *P*_50−75_, and >*P*_75_) of %BF in each sex.

Information on alcohol consumption was based on self-report. Alcohol drinking was classified into three groups: current drink, ex-drink, and never drink. The definition for each group of alcohol consumption has been described previously (13, 14). In brief, the current drink was defined as the consumption of at least 30 g of alcohol and had lasted for at least 6 months; quit drink was defined as stopped drinking for at least half 1 year. Alcohol consumption risk level was classified based on the WHO's guideline, which was defined as low (1–40 g/day), medium (41–60 g/day), and high (>60 g/day) in men, and 1–20, 21–40, and >40 g/day, respectively, in women (15).

### Other Covariates

Sociodemographic characteristics (e.g., age, sex, the highest educational attainment, living in urban or rural areas) were collected by standardized questionnaire interview. Cigarette smoking was grouped into three categories: current smoking, quit smoking, and never smoking. Current smoking was defined as smoking at least one cigarette per day for at least half 1 year. Quit smoking was defined as having quit tobacco use for more than 6 months preceding the survey (12). Glomerular filtration rate [eGFR, ml/min per 1.73 m^2^)] was calculated according to the Modification of Diet in Renal Disease equation for Chinese (c-MDRD) as follows (16):

(1) $$\begin{array}{r} eGFR\;=\;175\times Scr^{-1.234}\times age^{-0.179}\times 0.742\left(if\;female\right) \end{array}$$

### Statistical Analysis

The statistical analyses were performed using SAS version 9.4 for Windows (SAS Institute Inc, Cary, NC, USA). Continuous variables were presented as means and standard deviations (SDs) and categorical data as frequencies and percentages (%). Parings of BMI and %BF groups were formed. The expanded bodyweight categories are shown in Table 1. To address the aims of this study, we used QR, modeling SUA as the dependent variable. The QR models the median, rather than the mean of the outcome variable (SUA in this study), and any other percentile across the frequency distribution without the need to categorize participants. To compare the effect size of QR models with the general linear regression model (GLM), the mean estimates were also calculated and presented in the results, with adjusted covariates consistent with QR models. As no positive association was tested in both GLM and QR models between alcohol consumption and SUA among women, the results concerning such relationship were restricted only in men.

**Table 1:** Parings of BMI and %BF groups in the study population.

**BMI category**		**%BF quartile**	***N*** **(%)**	**BMI (kg/m^**2**^)**	**%BF**
Under/normal weight	NWBF1	Q1	7,527 (24.26)	19.87 ± 1.66	20.27 ± 6.11
	NWBF2	Q2	7,275 (23.45)	22.43 ± 1.16	26.56 ± 5.06
	NWBF3	Q3	4,759 (15.34)	23.89 ± 0.81	30.91 ± 4.91
	NWBF4	Q4	234 (0.75)	24.26 ± 0.89	31.91 ± 5.14
Overweight	OWBF2	Q2	351 (1.13)	25.66 ± 0.70	21.56 ± 2.27
	OWBF3	Q3	3,046 (9.82)	25.94 ± 0.79	28.22 ± 5.64
	OWBF4	Q4	6,249 (20.15)	27.50 ± 1.25	34.40 ± 5.95
Obesity	OBBF4	Q4	1,579 (5.09)	32.14 ± 2.17	38.48 ± 7.56

*BMI, body mass index; %BF, body fat percentage. Other categories were omitted because of few numbers*.

The final sample was 31,746, restricted to subjects without missing values of SUA, BMI or %BF, and eGFR ≥ 60 ml/min per 1.73 m^2^ [to exclude potential kidney disorders (17)], no self-reported as gout or using diuretic medication. The flowchart of the inclusion and exclusion criteria for the analytical sample was illustrated in our previous work (18). As SUA varies substantially by sex, we analyzed the data by sex separately. Due to few data in the lowest %BF with overweight, and the Q1–Q3 %BF with obesity groups, the final measurement of body adiposity only consisted of 8 groups (Table 1). A *p* < 0.05 (two-tailed) was considered as statistically significant.

## Results

Our primary analytical sample included 31,746 Chinese adults who aged 20–80 years; 40% were male and 64% were from urban areas. Men had a higher prevalence of overweight or obesity than women, and had a higher level of SUA level. The basic characteristics of participants are presented in Table 2.

**Table 2:** Basic characteristics of the study participants in CNHS.

	**Male (*****n*** **=** **12,701)**		**Female (*****n*** **=** **19,045)**		***p-*** **values**
**Age (year)**	48.99	13.46	48.27	13.14	<0.001
20-	1,309	10.31	2,048	10.75	<0.001
30-	2,052	16.16	3,144	16.51	
40-	3,233	25.45	5,136	26.97	
50-	3,184	25.07	4,749	24.94	
60-	2,156	16.98	3,019	15.85	
70–80	767	6.04	949	4.98	
**Residential areas**					
Urban	7,925	62.40	12,315	64.66	<0.001
Rural	4,758	37.46	6,694	35.15	
**Education**					
Illiterate/primary school	2,297	18.09	5,716	30.01	<0.001
Middle/high school	6,492	51.11	8,731	45.84	
College or above	3,881	30.56	4,549	23.89	
**Alcohol drinking**					
Current drinking	8,236	64.85	2,993	15.72	<0.001
Quit drinking	1,269	9.99	374	1.96	
Never drinking	3,159	24.87	15,638	82.11	
**Among current drinkers**					
Low risk drinking	7,492	78.52	2,896	85.00	<0.001
Moderate risk drinking	657	6.89	87	2.55	
High risk drinking	1,216	12.74	64	1.88	
%BF (%)	21.50	5.50	32.38	6.11	<0.001
BMI (kg/m^2^)	24.41	3.53	23.65	3.57	<0.001
**BMI categories**					
<25	7,231	56.93	12,798	67.20	<0.001
25-	4,615	36.34	5,175	27.17	
≥30	730	5.75	886	4.65	
**%BF categories**					
Q1	3,029	23.85	4,542	23.85	0.2706
Q2	3,043	23.96	4,592	24.11	
Q3	3,196	25.16	4,620	24.26	
Q4	3,177	25.01	4,890	25.68	
SUA (μmol/l)	366.93	85.61	277.76	66.89	<0.001
**SUA categories**					
P_5_	240.80	–	181.40	–	
P_25_	307.70	–	231.60	–	
P_50_	360.00	–	271.00	–	
P_75_	417.40	–	316.30	–	
P_95_	517.60	–	398.20	–	

*BMI, body mass index; %BF, body fat percentage (%); SUA, serum uric acid level; Q1–Q4, the first to fourth quartile of %BF; P_5_-P_95_, The 5th, 25th, 50th, 75th, and 95th percentile of SUA level (μmol/l)*.

### Body Adiposity and SUA

In the GLM estimations, overweight and obesity were strongly associated with higher SUA (all *p* < 0.001), but we found that these associations were stronger at the upper percentiles of SUA by QR estimations (Table 3). For instants, in obese men, the QR coefficients at the 75th and 95th percentiles were 74.0 (63.1–84.9) and 80.9 (52.5–109.3), respectively. Noticeable, in the linear regression model, the estimated coefficient for this relationship was 68.4 (62.3–74.6) for the entire men who were recruited. Similar situations were also found in the associations of %BF with SUA in both sexes (Table 3). The higher the %BF was, the larger the regression coefficients were. The highest quartile of %BF in men had a 92.6 (79.3–105.9) μmol/L higher SUA levels at its 95th percentile than the first quartile (*p* < 0.001).

**Table 3:** The association of excess bodyweight and alcohol consumption with SUA based on QR models.

**Exposure**	**GLM regression**	**5th**		**25th**		**50th**		**75th**		**95th**	
		**Beta**	***p***	**Beta**	***p***	**Beta**	***p***	**Beta**	***p***	**Beta**	***p***
**Male**											
**BMI category (ref** **=** **under/normal weight)**											
Obesity	68.4 (62.3–74.6)	52.8 (41.9–63.7)	<0.001	62.2 (53.9–70.5)	<0.001	67.5 (58.3–76.8)	<0.001	74.0 (63.1–84.9)	<0.001	80.9 (52.5–109.3)	<0.001
Overweight	38.3 (35.3–41.3)	24.3 (18.9–29.7)	<0.001	35.1 (31.3–39.0)	<0.001	39.7 (35.9–43.4)	<0.001	43.4 (39.1–47.7)	<0.001	43.1 (34.5–51.7)	<0.001
**%BF**											
Q2	25.2 (21.2–29.3)	16.0 (9.99–22.0)	<0.001	21.9 (17.2–26.6)	<0.001	26.6 (22.2–30.9)	<0.001	31.2 (26.2–36.1)	<0.001	30.2 (18.5–41.8)	<0.001
Q3	47.1 (43.0–51.1)	28.80 (22.7–34.9)	<0.001	40.6 (36.0–45.2)	<0.001	46.3 (41.7–50.9)	<0.001	55.1 (50.3–59.8)	<0.001	62.8 (50.6–75.0)	<0.001
Q4	71.6 (67.5–75.6)	46.5 (40.0–53.0)	<0.001	62.7 (57.8–67.6)	<0.001	70.0 (64.6–75.4)	<0.001	82.3 (76.1–88.6)	<0.001	92.6 (79.3–105.9)	<0.001
**Alcohol consumption (ref** **=** **never drink)**											
Ex-drinker	13.9 (8.6–19.3)	5.9 (−2.8–14.5)	0.182	8.6 (1.0–16.1)	0.026	13.5 (7.1–20.0)	<0.001	17.7 (9.4–26.0)	<0.001	30.1 (11.9–48.4)	0.001
Current drinker	21.2 (17.8–24.6)	14.5 (8.4–20.6)	<0.001	19.1 (15.2–23.0)	<0.001	21.0 (17.1–24.9)	<0.001	21.7 (16.5–26.9)	<0.001	31.8 (22.6–41.0)	<0.001
**Alcohol risk group among current drinkers (ref** **=** **never drink)**											
Low risk	17.4 (14.0–20.8)	12.2 (6.6–17.8)	<0.001	15.7 (12.1–19.3)	<0.001	17.8 (14.1–21.5)	<0.001	17.6 (12.2–23.0)	<0.001	26.2 (16.9–35.5)	<0.001
Moderate risk	27.4 (20.5–34.2)	20.6 (7.9–33.2)	<0.001	26.6 (16.6–36.6)	<0.001	25.2 (16.4–33.9)	<0.001	28.7 (18.0–39.4)	<0.001	40.7 (18.3–63.2)	<0.001
High risk	34.2 (28.8–39.5)	28.4 (19.3–37.4)	<0.001	28.5 (21.9–35.1)	<0.001	33.4 (26.3–40.4)	<0.001	36.2 (27.8–44.7)	<0.001	57.4 (42.5–72.3)	<0.001
**Female**											
**BMI category (ref** **=** **under/normal weight)**											
Obesity	55.0 (50.7–59.3)	41.1 (31.3–50.9)	<0.001	44.5 (39.1–49.9)	<0.001	55.9 (50.1–61.7)	<0.001	63.9 (54.8–73.0)	<0.001	79.9 (68.2–91.6)	<0.001
Overweight	31.9 (29.8–34.0)	20.2 (16.7–23.7)	<0.001	26.9 (24.3–29.5)	<0.001	31.7 (29.0–34.3)	<0.001	37.1 (34.0–40.2)	<0.001	45.7 (39.7–51.7)	<0.001
**%BF (ref** **=** **Q1)**											
Q2	18.2 (15.6–20.8)	11.6 (7.5–15.7)	<0.001	15.7 (13.3–18.1)	<0.001	17.0 (14.5–19.5)	<0.001	21.2 (17.9–24.5)	<0.001	28.0 (21.4–34.6)	<0.001
Q3	34.6 (31.9–37.2)	24.1 (19.9–28.3)	<0.001	28.6 (25.7–31.5)	<0.001	33.8 (30.7–36.9)	<0.001	40.4 (37.3–43.5)	<0.001	50.1 (41.0–59.3)	<0.001
Q4	58.7 (56.0–61.3)	36.3 (31.4–41.2)	<0.001	47.6 (44.6–50.6)	<0.001	56.8 (53.8–60.3)	<0.001	68.2 (64.1–72.3)	<0.001	90.3 (83.0–97.7)	<0.001

*SUA, serum uric acid; QR, quantile regression; GLM, general linear regression model; %BF, body fat percentage (%); Q1–Q4, the first to fourth quartile of %BF. The 5th, 25th, 50th, 75th, and 95th percentile of SUA in men are 240.8, 307.7, 360.0, 417.4, and 517.6 μmol/l, respectively, and are 181.4, 231.6, 271.0, 316.3, and 398.2 μmol/l in women, respectively. Beta, the regression coefficient*.

**Figure 1:**
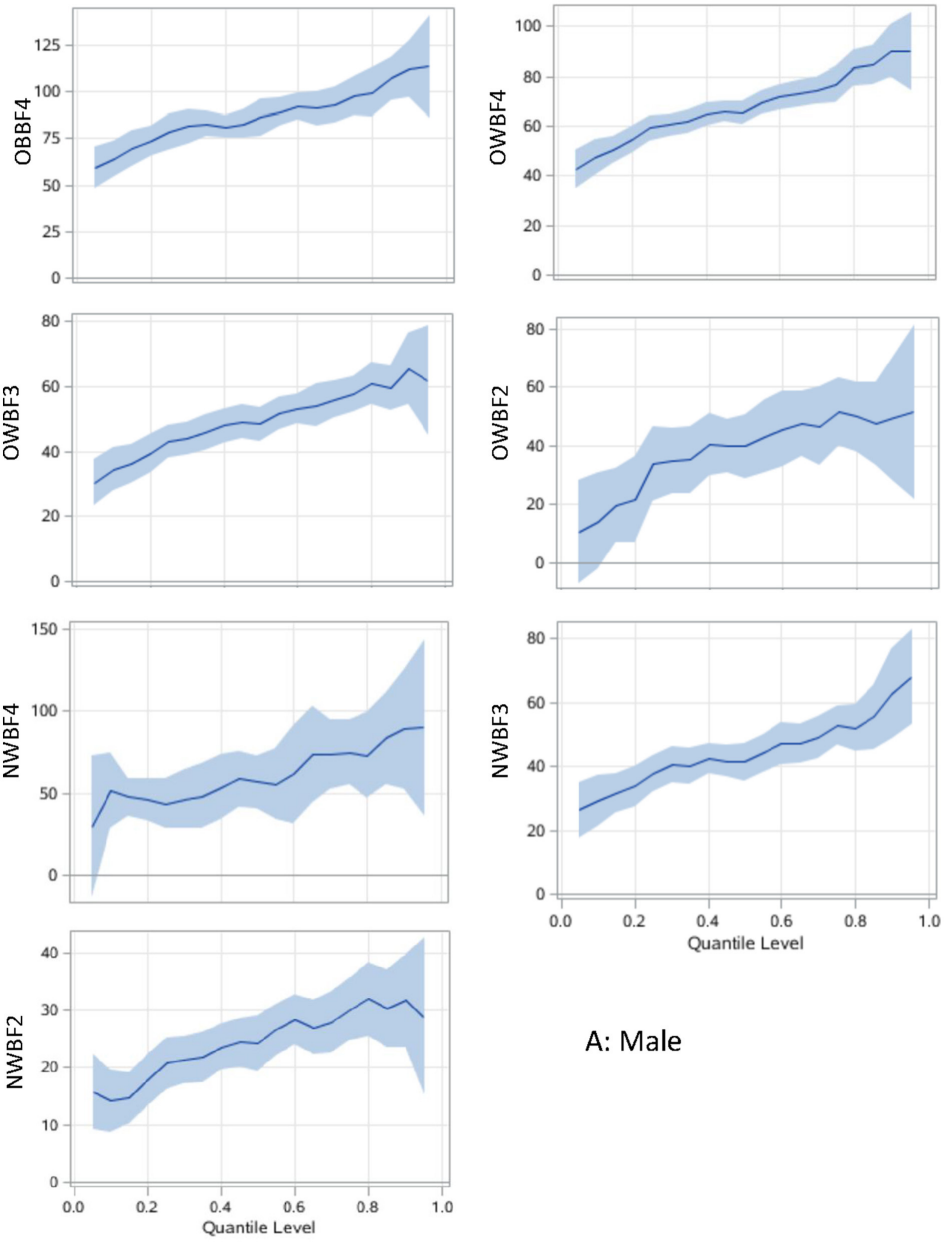
Associations between body adiposity and SUA among men using quantile regression models. The under or normal weight group with the lowest %BF quartile is the reference group. The models are adjusted for age, study sites, residential areas, educational attainment, and alcohol consumption. The x-axis is the quantile level of SUA, and the y-axis is the difference of SUA level between the current group and the reference group. The solid lines represent the estimated effect of the associations, and the shadows are their 95% confidence intervals. SUA, serum uric acid; %BF, body fat percentage; NWBF 2, under/normal weight with the second quartile of %BF; NWBF3, under/normal weight with the third quartile of %BF; NWBF4, under/normal weight with the fourth quartile of %BF; OWBF2, overweight with the second quartile of %BF; OWBF3, overweight with the third quartile of %BF; OWBF4, overweight with the fourth quartile of %BF; OBBF4, obesity with the fourth quartile of %BF.

**Figure 2:**
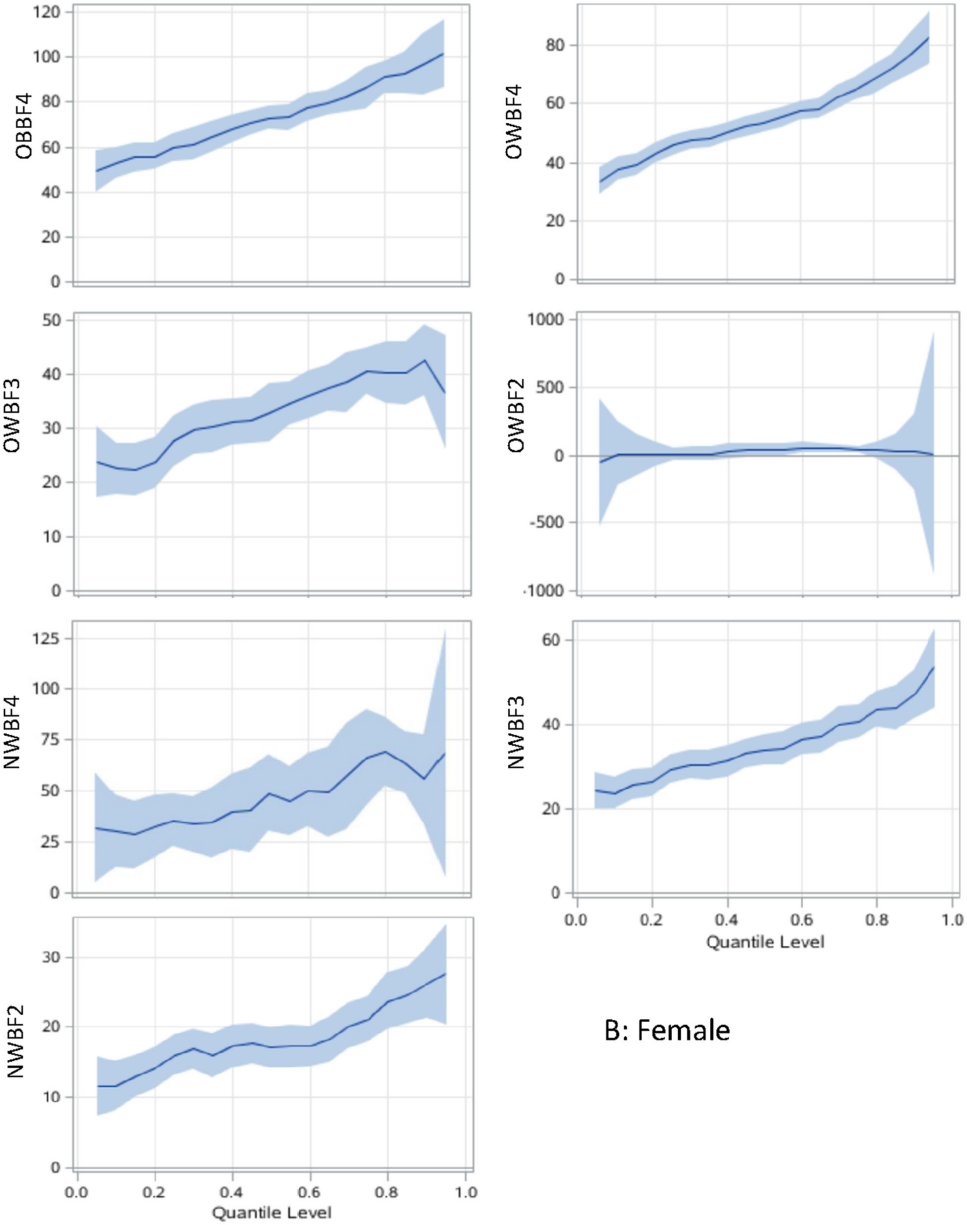
Body adiposity associations with SUA among women using quantile regression models. The under/normal weight group with the lowest %BF quartile is the reference group. The models are adjusted for age, study sites, residential areas, educational attainment, and alcohol consumption. The x-axis is the quantile level of SUA, and the y-axis is the difference of SUA level between the current group and the reference group. The solid lines represent the estimated effect of the associations, and the shadows are their 95% confidence intervals. SUA, serum uric acid; %BF, body fat percentage; NWBF2, under/normal weight with the second quartile of %BF; NWBF3, under/normal weight with the third quartile of %BF; NWBF4, under/normal weight with the fourth quartile of %BF; OWBF2, overweight with the second quartile of %BF; OWBF3, overweight with the third quartile of %BF; OWBF4, overweight with the fourth quartile of %BF; OBBF4, obesity with the fourth quartile of %BF.

The associations between paring of body adiposity categories and SUA are presented in Figures 1, 2 by sex. Compared with normal or underweight with the lowest %BF group (NWBF1), the obesity-highest %BF group (OBBF4) had the strongest positive effect on SUA, especially at the higher percentile of SUA (Figure 1).

The levels of body adiposity categories were positively associated with SUA when were analyzed using GLM (all *p* < 0.001), whereas the association occurred a different way when QR models were fitted. In overweight participants (OWBF2–OWBF4, Figure 1), the %BF quartile was positively associated with SUA level, but with varied effect size at different SUA percentiles. The highest quartile of %BF was positively associated with all SUA percentiles in both sexes, but no statistically significant association was found among lower SUA percentile in the second and third quartile of %BF (Figures 1, 2). Similarly, in normal or underweight participants (NWBF1–NWBF4), a higher quartile of %BF had a greater effect size in all SUA percentiles. %BF was more likely to affect SUA, as among men, in overweight with the second %BF quartile (OWBF2) group, the QR coefficient was smaller than in the NWBF3 (under/normal weight with the third %BF quartile) group in almost all SUA percentile, and also smaller in the NWBF4 (under/normal weight with the fourth %BF quartile) group (Figure 1). Noticeably, although defined as “normal weight,” higher %BF level also led to elevated SUA, as in the NWBF4 group, the QR coefficient was >50 at the median percentile.

### Alcohol Consumption and SUA

**Figure 3:**
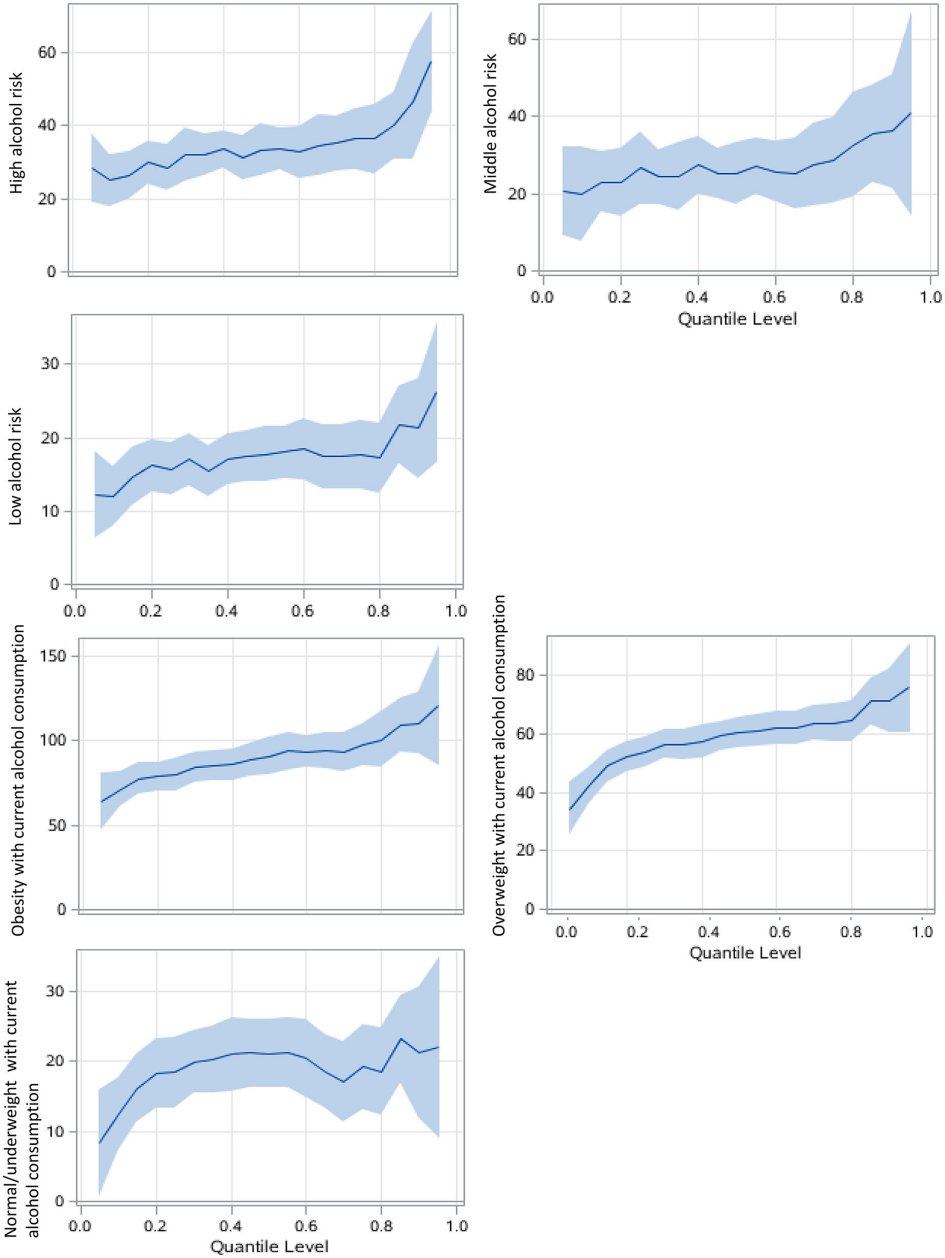
Alcohol consumption associations with SUA among men using QRs. The never drink group was the reference group. The models focusing on alcohol risk levels are adjusted for age, study sites, residential areas, educational attainment, and body adiposity categories. The BMI-alcohol consumption combined models are adjusted for covariates similarly except for body adiposity. The x-axis is the quantile level of SUA, and the y-axis is the difference of SUA level between the current group and the reference group The solid lines represent the estimated effect of the associations, and the shadows are their 95% confidence intervals.

In the association between alcohol consumption and SUA level, current alcohol consumption had a greater effect size in both GLM and QR models than ex-drinkers and never-drinkers (Table 3). Current alcohol drinking had the strongest effect at the 95th percentile of SUA (regression coefficient: 31.8, 95% CI: 22.6–41.0, compared with 14.5, 95% CI of 8.4–20.6 in the 5th percentile). The high risk of alcohol consumption had a greater effect on SUA, especially in higher SUA percentile (Table 3, Figure 3). Compared with the general effect estimated by the GLM (with a regression coefficient of 34.2), the high-risk group had 57.4 μmol/L (42.5–72.3) higher SUA levels than the never-drink group in the 95th percentile of SUA.

Notably, overweight or obese participants who currently drink had much larger regression coefficients than their counterparts, and in the higher percentile, that is, over 80th percentile of SUA, the coefficient was larger than 100 in the obese with current alcohol consumption group, compared with never drinker with under or normal weight (Figure 3).

## Discussion

In this study, for the first time, we investigated the association among body adiposity, alcohol consumption, and SUA using QR analysis in a large general Chinese population. The results showed that adiposity and alcohol consumption are related to a higher level of SUA, especially at its upper percentile distribution. It is suggested that current interventions, such as weight loss or alcohol intake reduction, which targets on lowing SUA level, may achieve better effect in population with much higher SUA levels.

Previous studies have reported the association among body adiposity, alcohol consumption, and SUA in different populations. Overweight and obesity are important risk factors in the development of both hyperuricemia and gout (19). Data from the China Health and Retirement Longitudinal study indicated that for both men and women, obesity is a risk factor for hyperuricemia (20). A study in Henan Province of China also revealed that overweight or obesity could increase the risk of hyperuricemia (21). Han et al. found that in an eastern Chinese population, compared with normal-weight people, overweight and obese subjects had higher SUA levels and hyperuricemia prevalence (22). Furthermore, the association between hyperuricemia and obesity has been found to have a temporal relationship, and the comorbidity of them may lead to a higher risk of other cardiovascular diseases (23). A secondary analysis of the Dietary Intervention Randomized Controlled Trial (DIRECT) study (24) revealed that weight-loss interventions can lower the SUA level by lowing BMI. In this study, bodyweight measured by BMI was found to be strongly associated with SUA, but the effect size was stronger in male than in female, which revealed by both GLM and QR models. This is not consistent with some previous studies (20). The possible reason that explains this sex-specific difference may be due to the sex-specific physiologic impact of SUA, or the difference between the severity of other mediators influencing both bodyweight and SUA. Gonadal hormone levels are linked with age and may have an impact on the development of metabolic diseases (25). The prevalence of hyperuricemia in postmenopausal women would increase (21, 26) which indicated that gonadal hormone may also play a role in the etiology of elevated SUA in women. To test this hypothesis, we did further analysis that included only participants aged over 60 and then found out that the effect size of bodyweight (measured by BMI) on SUA in male and female were was similar. This finding also suggested that bodyweight intervention to lowing SUA is more important in postmenopausal women.

Only few studies explored the relationship between body fat and SUA in restricted populations. For instants, Niu et al. reported that SUA is associated with adiposity factors, especially with fat mass reduction in obese children and adolescents (27), and Cremonini et al. observed that %BF was associated with increased SUA level in postmenopausal women (28).

To our knowledge, limited study has explored the relationship between the combination of bodyweight and %BF and SUA. As calculated by height and weight, BMI does not allow the identification of fat-free mass from adipose tissue (29) and thus may misclassify individuals with excess body fat as normal or healthy (30). In this study, we classified body adiposity under consideration of both BMI and %BF, that is, the expanded bodyweight categories, to better estimate how adiposity would influence SUA. We found that compared with BMI, the association between body fat and SUA seemed stronger. As demonstrated by the QR models, higher bodyweight with lower %BF had a smaller effect size than low bodyweight with higher %BF. Elevated SUA levels may associated with fat accumulation through multiple pathways, such as increasing uric acid-dependent intracellular and mitochondrial oxidative stress (31), activating the nuclear transcription factor, carbohydrate-responsive element-binding protein, and increased ketohexokinase expression (32), or inhibition of AMP-activated protein kinase (33). Previous population-based studies have suggested that weight loss is related to decreased SUA (24, 34). Our study suggested that population with the peak level of SUA may benefit more by weight loss, thus preventing further health hazards caused by extremely high levels of SUA.

Alcohol intake has been found to be associated with elevated SUA. For instants, revealed by the Third National Health Nutrition Examination Survey (NHANES III), alcohol intake (especially beer and liquor) was positively associated with SUA level (35). A study in Brazilian also supported that alcoholic beverage was associated with increased SUA (36). The mechanism of this association may be explained by that ethanol intake could increase SUA via either increased urate production resulting from enhanced turnover of adenine dinucleotide phosphate, or decreased renal excretion of uric acid which is the secondary to lactate levels, whereby lactate can competitively inhibit urate secretion by the proximal tubule (37–39). As demonstrated by our findings, alcohol consumption, especially with higher daily alcohol intake, has a stronger effect on SUA level, particularly at the distribution of over 80th percentile (the regression coefficients were over 50), but this effect cannot be explored using traditional GLM method. People who quit alcohol drinking also had a higher level of SUA than never drinkers, probably because some people quit alcohol consumption because of illness, such as type 2 diabetes (40), which is also highly associated with elevated SUA (41).

The obese men who currently drink alcohol had the highest SUA levels than their counterparts. The combination of two risk factors, that is, alcohol consumption and adiposity, may lead to worse health outcomes. As indicated by our results, the regression coefficients among obese men who currently drink alcohol are greater than the combination of the effect size of single obese or currently drinking. Shiraishi and Une also reported in a Japanese male population that the combined risk was greater than the sum of the effect of obesity and drinking on hyperuricemia (42). Therefore, an intervention package covering both alcohol reduction and weight loss may achieve better health outcome in a vulnerable population of hyperuricemia.

The strength of this study is first in its large representative sample size. With a multilevel stratified sampling method, we selected our participants from different geographic areas of China, covering both urban and rural areas. Second, in this study, we used QR modeling methodology instead of ordinary linear squares (OLS) regression, and thus, the results are more robust and comprehensive than only the mean estimates which are commonly more sensitive to outliers and can be influenced by imbalances of extreme data in the upper or lower tails of the distribution of outcome (43). Last but not the least, the quality of data collection guaranteed the reliability of health estimates in the first place. In the survey, we developed strict quality control measures. For example, completeness and rationality of each questionnaire were examined by an epidemiologist by face-to-face recheck with the participant; fixed staff were assigned to perform the body composition measurement, etc. (12).

The limitations of this study should also be acknowledged. As the nature of cross-sectional design, we cannot conclude a causal relationship among adiposity, alcohol consumption, and elevated SUA. Besides, the information of alcohol intake was just recorded once, ignoring the variation in alcohol consumption over time. Third, we did not collect dietary information which may play important role in influencing SUA level, thus limiting our further investigation on the interaction effect between dietary intake and body adiposity on SUA. Fourth, the sample size in some subgroups, for example, the OWBF 2 group, is relatively small, which resulted in unstable effect estimates.

In summary, the observation of stronger association at the higher percentile of SUA suggests that decreasing excess body adiposity and alcohol intake at the populational level may shift the upper tails of the SUA distributions to lower values, thereby reducing the incidence of elevated SUA. For public health policymakers, healthcare providers, the findings of the study could provide new knowledge on how lifestyle intervention would influence the health indexes at different levels and help to identify the priority health care needs.

## Data Availability Statement

The original contributions presented in the study are included in the article/supplementary material, further inquiries can be directed to the corresponding author.

## Ethics Statement

The studies involving human participants were reviewed and approved by the Bioethical Committee of Institute of Basic Medical Sciences, Chinese Academy of Medical Sciences. The patients/participants provided their written informed consent to participate in this study.

## Author Contributions

HH and GS conceptualized the study, carried out data curation, and provided funding acquisition. HH performed the methodology and formal analysis, involved in writing—original draft preparation, and provided the software. HH, GS, and LPan validated the manuscript and conducted project administration. LPan, XR, DW, JD, ZC, HW, XW, FL, LPa, XP, YW, CY, GS, and HH conducted the investigation. GS, HH, XR, DW, JD, ZC, HW, XW, FL, LPa, XP, YW, and CY provided the resources. GS done writing, reviewing, and editing. HH, YW, and CY performed visualization. GS and LPan supervised the study. All authors read and approved the final manuscript.

## Funding

This work was supported by the National Key R&D Program of China (Grant No. 2016YFC0900600/2016YFC0900601), the National Natural Science Foundation of China (Grant No. 82003531), the Key Basic Research Program of the Ministry of Science and Technology of China (Grant No. 2013FY114100), and the CAMS Innovation Fund for Medical Sciences (CIFMS) (Grant No. 2020-I2M-2-009).

## Conflict of Interest

The authors declare that the research was conducted in the absence of any commercial or financial relationships that could be construed as a potential conflict of interest.

## Publisher's Note

All claims expressed in this article are solely those of the authors and do not necessarily represent those of their affiliated organizations, or those of the publisher, the editors and the reviewers. Any product that may be evaluated in this article, or claim that may be made by its manufacturer, is not guaranteed or endorsed by the publisher.
